# Assessing *Onchocerca volvulus* Intensity of Infection and Genetic Diversity Using Mitochondrial Genome Sequencing of Single Microfilariae Obtained before and after Ivermectin Treatment

**DOI:** 10.3390/pathogens12070971

**Published:** 2023-07-24

**Authors:** Shannon M. Hedtke, Young-Jun Choi, Anusha Kode, Gowtam C. Chalasani, Neha Sirwani, Stephen R. Jada, An Hotterbeekx, Michel Mandro, Joseph N. Siewe Fodjo, Glory Ngongeh Amambo, Raphael A. Abong, Samuel Wanji, Annette C. Kuesel, Robert Colebunders, Makedonka Mitreva, Warwick N. Grant

**Affiliations:** 1 Department of Environment and Genetics, School of Agriculture, Biomedicine and Environment, La Trobe University, Bundoora, VIC 3086, Australia; a.kode@latrobe.edu.au (A.K.); gchalasani@illumina.com (G.C.C.); n.sirwani@latrobe.edu.au (N.S.); w.grant@latrobe.edu.au (W.N.G.); 2 Department of Medicine, Washington University in St. Louis and McDonnell Genome Institute, St. Louis, MO 63108, USA; choi.y@wustl.edu (Y.-J.C.); mmitreva@wustl.edu (M.M.); 3 Amref South Sudan, Juba P.O. Box 30125, South Sudan; stephen.jada@amref.org; 4 Global Health Institute, University of Antwerp, Doornstraat 331, 2610 Antwerp, Belgium; an.hotterbeekx@uantwerpen.be (A.H.); josephnelson.siewefodjo@uantwerpen.be (J.N.S.F.); robert.colebunders@uantwerpen.be (R.C.); 5 Provincial Health Division Ituri, Ministry of Health, Bunia P.O. Box 57, Democratic Republic of the Congo; michelmandro8@gmail.com; 6 Parasites and Vectors Research Unit, Department of Microbiology and Parasitology, Faculty of Science, University of Buea, Buea P.O. Box 63, Cameroon; gloryngongeh@yahoo.com (G.N.A.); abongr@gmail.com (R.A.A.); samwandji@gmail.com (S.W.); 7 Research Foundation for Tropical Diseases and Environment (REFOTDE), Buea P.O. Box 474, Cameroon; 8 UNICEF/UNDP/World Bank/World Health Organization Special Programme for Research and Training in Tropical Diseases (TDR), World Health Organization, 1202 Geneva, Switzerland; kuesela@who.int

**Keywords:** onchocerciasis, population genetics, microfilariae, epidemiology, macrofilariae, elimination, monitoring, drug studies

## Abstract

Onchocerciasis is a neglected tropical disease targeted for elimination using ivermectin mass administration. Ivermectin kills the microfilariae and temporarily arrests microfilariae production by the macrofilariae. We genotyped 436 microfilariae from 10 people each in Ituri, Democratic Republic of the Congo (DRC), and Maridi County, South Sudan, collected before and 4–5 months after ivermectin treatment. Population genetic analyses identified 52 and 103 mitochondrial DNA haplotypes among the microfilariae from DRC and South Sudan, respectively, with few haplotypes shared between people. The percentage of genotype-based correct assignment to person within DRC was ~88% and within South Sudan ~64%. Rarefaction and extrapolation analysis showed that the genetic diversity in DRC, and even more so in South Sudan, was captured incompletely. The results indicate that the per-person adult worm burden is likely higher in South Sudan than DRC. Analyses of haplotype data from a subsample (*n* = 4) did not discriminate genetically between pre- and post-treatment microfilariae, confirming that post-treatment microfilariae are not the result of new infections. With appropriate sampling, mitochondrial haplotype analysis could help monitor changes in the number of macrofilariae in a population as a result of treatment, identify cases of potential treatment failure, and detect new infections as an indicator of continuing transmission.

## 1. Introduction

Over 120 million people are at risk of contracting onchocerciasis, a neglected tropical disease (NTD) caused by the filarial nematode *Onchocerca volvulus* and transmitted by blackflies in the genus *Simulium*, primarily in sub-Saharan Africa [1]. Onchocerciasis pathology includes skin depigmentation and lesions, ocular damage that can lead to blindness, and neurological–endocrine disorders such as epilepsy and Nakalanga syndrome [2,3,4]. The World Health Organization (WHO), in conjunction with government and non-government agencies, are targeting the elimination of *O. volvulus* transmission in endemic countries [5]. The strategy for interrupting transmission is the mass drug administration of ivermectin (MDAi). Ivermectin kills the larval stages (microfilariae) found in the skin of infected persons (microfilaricidal effect), and temporarily stops female adult worm reproduction (embryostatic effect). It is distributed to affected communities annually, biannually, or even quarterly. With each subsequent exposure to ivermectin, the fertility of female worms may be reduced, although the estimated degree of this cumulative effect ranges across studies [6,7,8,9]. The effects of long-term MDAi on skin microfilariae density and macrofilariae fertility have resulted in the WHO-verified elimination of transmission in most endemic areas in the Americas and may have also resulted in elimination in several endemic areas in sub-Saharan Africa [10,11,12,13,14]. These successes motivated shifting the goal from control as a public health problem to the elimination of onchocerciasis transmission [5,15].

One challenge facing the elimination of the transmission of *O. volvulus* with MDAi in sub-Saharan Africa is the variable response of adult worms to the embryostatic effect of ivermectin, with increases in skin microfilariae density post treatment beginning as early as a few weeks or as late as >1 year [16,17,18,19,20,21,22,23,24,25]. Repopulation of the skin with microfilariae 80 days post treatment, or the observation of stretched microfilariae in the uteri of adult female worms 80–90 days post treatment, has been referred to as a sub-optimal response (SOR) to ivermectin [16,17,18,19,20,21,22,23,24,26]. In addition, a sub-optimal microfilariae response (SOMR) has been described, in which the rate or extent of the initial reduction in skin microfilariae following ivermectin treatment is reduced [24,25,27]. The microfilaricidal and embryostatic effect of ivermectin may differ between geographic areas [20,24]. Thus, tracking community-specific changes in microfilaridermia and worm fertility is useful for the quantitative assessment of the effect of MDAi and/or for parameterizing models used to predict the optimal timing and duration of MDA for the successful elimination of transmission [28,29].

Current parasitological methods for assessing the effect of MDAi on an individual or community level [16,17,21,22] do not allow the estimation of the number of fertile adult worms in a host or in a community. Palpable subcutaneous nodules containing adult worms can be excised for analysis, but an unknown (and significant) number of worms are inaccessible deep in the body [30,31,32,33,34], and the number of individuals who can be sampled is limited by the fact that minor surgery is required. Estimates of the number of live female adult worms per person living in the West African savannah based on the statistical analyses of macrofilariae in excised nodules indicate high variability among hosts within the same community, ranging from approximately 4 to 177 [35,36] worms per person. 

Our goal is to develop a tool that can quantitively measure progress towards reducing the number of fertile worms during MDAi (and complementary interventions). Because maternal sibling microfilariae sampled from the same person will have identical mitochondrial DNA sequences (or haplotypes) due to the strictly maternal nature of mitochondrial inheritance, we can estimate how many adult females (macrofilariae) are reproductively active by counting the number of unique mitochondrial haplotypes identified in the microfilariae sampled from the skin, assuming sister nematodes rarely infect the same person. Because a particular skin snip may not contain microfilariae offspring from all female worms in the person, the number of haplotypes detected will represent the minimum number of reproductively active female worms. However, assuming that the subset represents a random sample of the microfilariae in the host, changes over time in the number of haplotypes estimated should indicate whether MDAi is effectively reducing the number of reproductively active worms. More explicitly, the number of haplotypes present amongst the microfilariae should decline, as MDAi reduces transmission (and thus new infections) and adult females reach the end of their reproductive life span, thus providing a measure of progress towards the endpoint of zero fertile adult females.

We opportunistically utilized microfilariae collected in two studies whose focus was on onchocerciasis-associated epilepsy, one in the Logo Health zone in the Ituri province of the Democratic Republic of the Congo (DRC) and the other in Maridi county of South Sudan. People enrolled in the studies had epilepsy (potentially onchocerciasis-associated epilepsy) and were ivermectin-naïve in the Logo Health zone [37]. To estimate how many microfilariae per person would need to be sequenced to determine the minimum number of reproductively active adult female worms, we performed rarefaction and extrapolation analysis of mitochondrial haplotype diversity. We compared these estimates between sampling locations to explore differences in baseline infection intensity. We compared haplotype diversity between the samples from the two countries and between microfilariae obtained pre- and post-treatment for four people from whom both pre- and post-ivermectin microfilariae were available. Finally, we placed mitochondrial haplotype diversity identified in the microfilariae from DRC and South Sudan in the context of sequence data from parasites from other endemic areas in sub-Saharan Africa.

## 2. Materials and Methods

### 2.1. Origin of Microfilariae 

Skin snips were collected from people with epilepsy in Maridi County in South Sudan and in the Logo Health Zone, Ituri Province, DRC in 2018 during epidemiological studies to investigate the association between onchocerciasis and epilepsy. Two snips were obtained from each participant at two time points: immediately before ivermectin treatment (“pre-treatment”) and 4 months (DRC) or 5 months (South Sudan) later (“post-treatment”). After the 24-h incubation of the snips in isotonic saline, the microfilaridermia was quantified microscopically [38,39,40] and samples were transferred to 80% ethanol and sent to La Trobe University, Victoria, Australia. In Maridi County, annual MDAi had been instituted since the early 2000s, but was interrupted for several years because of insecurity, and only reintroduced in 2017 with very low coverage (40.8%) [41]. At the time the skin snip was taken, each participant had either never taken ivermectin or had taken it only once. In the Logo Health zone, DRC, MDAi had never been instituted. A recent study concluded that today, *Simulium dentulosum* appears to be the main vector of human onchocerciasis in the area, and that *Simulium vorax* may be a secondary vector [42]. Ov16 IgG4 antibody positivity, determined with the Ov16 IgG4 Bioline rapid diagnostic test [43,44,45,46], between 2016 and 2018 was 0% (0/55) among 6-year-old and 7.1% (13/182) among 7–10-year-old children in the Logo Health zone [47]. In contrast, the Ov16 seroprevalence, determined with the same rapid test in an area in Maridi close to the blackfly breeding site, was 40% among children 3–6 years old and 66.7% among children 7–9 years old [48]. This suggests much higher ongoing *O. volvulus* transmission in Maridi compared to the Logo Health zone.

### 2.2. Origin of Adult Worms and Adult Worm Sequences

In 2016–2017, 27 adult female worms from the Centre Region and 12 from the Littoral Region in Cameroon were excised from nodules, as described in [20], and the heads (i.e., without uterine tissue) were placed in RNAlater (Invitrogen, Thermo Fisher Scientific, Waltham, MA, USA). 

Short-read data from adult worms were downloaded from the NCBI database from Benin, Côte d’Ivoire, Ghana, Guinea, Liberia, Mali, Sierra Leone, Liberia, Mali, and Uganda, project numbers PRJNA997216, PRJNA289926 [49], and PRJNA560089 [50].

### 2.3. DNA Extraction, Amplification and Sequencing

To minimize the transfer of host skin cell debris during DNA extraction, the microfilariae-containing ethanol solution was washed with Milli-Q^®^ water (Merck, KGaA, Darmstadt, Germany) by carefully transferring the ethanol solution containing the microfilariae, but without skin snip, into a 15 mL falcon tube, filling to 15 mL with Milli-Q water, and centrifuging at 500× *g* for 5 min. Approximately 14 mL was aspirated carefully from the tube, leaving the microfilarial pellet in approximately 1ml of now diluted ethanol solution. This was transferred into a clean reusable glass petri dish (glass because microfilariae are less adhesive to glass compared to plastic). Each microfilaria was picked by aspiration using a pipette (0.1–2 µL) under a dissecting microscope into 20 µL lysis buffer (10mM Tris–HCl, pH 8.0; 1mM EDTA, pH 8.0; add 1% Tween^®^20 (Sigma-Aldrich, Burlington, MA, USA) and Proteinase K 300 µg/mL (recombinant, PCR Grade, Sigma-Aldrich) added just prior to use). The transfer of any visible cell debris was avoided while picking microfilariae. Tubes each containing a microfilaria in lysis buffer were incubated at 55 °C for 2 or 3 h, followed by heat inactivating the solution at 80 °C or 85 °C for 20 min. DNA from adult worms from Cameroon was extracted using the Isolate II Genomic DNA kit (Bioline, London, UK) as per the manufacturer’s instructions.

Quantitative PCR (qPCR) was performed on the microfilarial lysates to confirm the presence of mitochondrial DNA prior to whole genome amplification. qPCR reactions were performed using 2 µL of 1:5 diluted microfilarial lysates, 5 µL of SsoAdvanced Universal SYBR Green master mix (Bio-Rad Laboratories, Hercules, CA, USA), 2 µL of nuclease-free water, and 0.5 µL each of 10 µM forward (SP-Ov-mt-10062: 5′-attggtgaccaataaccttca-3′) and reverse (ASP-Ov-mt-10062: 5′-ttgattcaatatcagggacgta-3′) primers. A synthesized 68-bp oligonucleotide (ttg att caa tat cag gga cgt ata ttt cgt caa tct gag ttg act ttg aag gtt att ggt cac caa t) was used as a positive control and standard and HPLC water as a negative control for all reactions. Oligonucleotides were synthesized by Integrated DNA Technologies (Redwood City, CA, USA). qPCR assays were run on a CFX Real-Time System (Bio-Rad Laboratories), with an initial denaturing step of 3 min at 98 °C followed by 40 cycles (of 98 °C 10 s, 54 °C 15 s, 72 °C 15 s, read plate) including melt curve analysis at 65 °C to 95 °C for 5 s with an increment of 0.2 °C for 5 s. Each of the diluted lysates was assayed in duplicate, and the standards in triplicate. The overall statistics for the qPCR runs were assessed using the CFX Maestro Software (Bio-Rad Laboratories). The cycle threshold (Cq) values for each sample were determined as positive if the Cq < 30, and negative if Cq > 30 [51,52].

Microfilariae are relatively small in size (250–360 × 5–9 µm [29]), with low yield of DNA from a single microfilaria. A minimum amount of starting DNA is prerequisite to the successful generation of Illumina sequencing libraries. Whole genome amplification (WGA) was used as an intermediate step on microfilarial DNA lysates to generate high yields of amplified DNA as required for library construction. To generate a sufficient quantity of microfilarial DNA for Illumina library construction, high-fidelity, multiple displacement whole genome amplification (WGA) was performed on each microfilaria using 2 µL lysate as the starting material and processed according to the manufacturer’s instructions (REPLI-g, QIAGEN GmbH, Hilden, Germany). The WGA reactions were performed at 30 °C for 16 h and heat-inactivated at 65 °C for 10 min. Concentrations of the WGA DNA were determined using a Qubit 2.0 fluorometer (Thermo Fisher Scientific, Waltham, MA, USA).

Sequencing libraries were constructed on samples whose DNA concentrations were above 1.0 ng/µL, using the Nextera DNA Flex/Illumina DNA prep library construction kits and barcoded using unique dual indexes, according to the manufacturer’s instructions (Illumina, San Diego, CA, USA). An Agilent TapeStation analysis was performed on libraries using D1000 ScreenTapes (Agilent Technologies, Santa Clara, CA, USA) to ensure that the libraries were within the selected size range of 400–650 bp. The Qubit fluorometer was used to quantify the libraries for pooling. The final 4nM pooled library was spiked with 1% PhiX control (Illumina) and run on a NovaSeq SP, 300 cycles (i.e., 150 bp paired reads) at the Australian Genomic Research Facility (Melbourne, Australia) or on a NextSeq 500, 300 cycles, at the La Trobe Genomics Platform (Bundoora, Australia).

### 2.4. Variant Calling

Raw reads were trimmed using *trimmomatic* v.0.32 [53]. Trimmed reads were competitively mapped to the *O. volvulus* nuclear and mitochondrial genomes v.4 [54], the *Wolbachia* bacterial endosymbiont (GenBank accession number NZ_HG810405.1), and *Homo sapiens* (genome GRCh38.p13) using *bwa* v.0.7.17 [55,56]. Reads that mapped to *H. sapiens* were counted for quality control assessment (Supplementary Table S1) and discarded from the data using Unix command line programs *grep* and *awk*. *Samtools* v.1.9 [57] was used to remove secondary and supplementary reads, and only reads that mapped uniquely to *O. volvulus* were retained. If a microfilaria was sequenced in more than one experiment, the mapped reads were combined. Depth was assessed using *bedtools* v.2.26.0 [58] and coverage for each *O. volvulus* chromosome at a depth of at least 20× was estimated using a custom Perl script (Supplementary Table S1). Samples that did not meet a minimum coverage of 85% at a depth of 20× were not included in the downstream analyses. *RepeatScout* v1.0.5 [59] was used to identify repetitive regions across *O. volvulus* chromosomes.

For sequences from adult worms, variants were called on the mitochondrial genomes using *GATK* v.4.0.11 *HaplotypeCaller* using a minimum read quality filter of 30. For sequences from microfilariae, *GATK* v.4.2.6.1 was used, with de bruijn graph on. Variants for each sample were combined and *GATK GenotypeGVCFs* was used for final variant calling. These data were then filtered using *GATK VariantFiltration* for quality and depth (-filter “QD < 2.0” --filter-name “QD2” -filter “QUAL < 30.0” --filter-name “QUAL30” -filter “SOR > 3.0” --filter-name “SOR3” -filter “FS > 60.0” --filter-name “FS60” -filter “MQ < 40.0” --filter-name “MQ40” -filter “MQRankSum < −12.5” --filter-name “MQRankSum-12.5” -filter “ReadPosRankSum < −8.0” --filter-name “ReadPosRankSum-8” -filter “DP > 20” --filter-name “DP20”).

Variants were also called using *freebayes* v.1.0.2 [60], and filtered for quality and depth using *vcftools* v.0.1.13 [61]. Variant calls were normalized using *bcftools* v.1.2 [62] and haplotypes were simplified using the vcfallelicprimitives function in *vcflib* [63]. *Bcftools* was then used to find the intersection between the calls made by *GATK* and those made by *freebayes*. Finally, *vcftools* was used to further filter the dataset to remove individuals with less than 99% of variants called, to remove sites with missing data, and to identify and remove indels, singletons, and repeat regions.

### 2.5. Data Analysis

The R package *adegenet* v.2.1.5 was used to produce principal component analysis (PCA) plots of the genetic diversity within people at each of the different collection time points and to cluster the worms [64,65]. Discriminant analysis of principal components (DAPC [66]), which maximizes the differences between predefined groups, was used to estimate the probability that a microfilaria would be correctly identified to person or to date of collection based on genotype. The number of PCs to use in DAPC was estimated using cross-validation. 

Rarefaction curves using microfilarial haplotypes from people from whom more than 10 microfilariae were successfully sequenced were produced using the R package *iNEXT* v.3.0.0 [67]. *Vegan* v.2.5–5 [65,68] was used to produce extrapolated estimates of total haplotype richness and associated standard error based on the Chao and abundance-based accumulated estimators, adjusted for sample bias [69,70,71].

*SNPEff* v.4.1l [72] was used to assess whether variants were in coding or noncoding regions and to calculate their effects on known genes (e.g., amino acid changes).

*PGDSpider* v.2.1.1.3 for Windows [73] was used to convert vcf to nexus format, which was then edited to add a “trait” block indicating person or country of origin. *PopArt* v.1.7 [74] was used to generate haplotype networks using the TCS approach [75], calculate diversity statistics, and to perform an analysis of molecular variance (AMOVA; [76]). 

## 3. Results

### 3.1. Sequencing Results

Out of 804 sequenced in total, 225 microfilariae from 10 people from the DRC and 211 microfilariae from 10 people from South Sudan successfully passed the filtering criteria and were included in the analyzed data (Table 1). Of the successfully sequenced microfilariae from the DRC, 207 were collected before treatment (D0); 15 and 3 microfilariae collected four months post treatment, were, respectively, from two persons from whom we do not have pre-treatment mtDNA sequences. The dataset from South Sudan includes 163 microfilariae collected before treatment and 48 collected around five months post treatment, with four people having parasites successfully sequenced from both pre- and post-treatment microfilariae samples.

### 3.2. Genetic Variation of Microfilariae within People

After merging and filtering, 439 variants (places in the genome where the base differs from the reference sequence) in the 13,744 bp mitochondrial genome were called, of which 119 were singletons (i.e., found in only one worm). Because singletons could be due to experimental error (e.g., the introduction of variation during whole genome amplification) rather than biological variation, we excluded these when determining the number of mitochondrial haplotypes represented in each sample, leaving 320 variants.

The number of mitochondrial haplotypes identified for the microfilariae from a person represents the minimum number of reproductively active female worms in that person (Table 1), since there may be unidentified haplotypes from microfilariae either not present in the skin snip, lost during transfer of the skin snip to ethanol, or not successfully sequenced. 

**Table 1:** Number of *Onchocerca volvulus* mitochondrial haplotypes estimated per person in the study areas in the Democratic Republic of Congo (DRC) and South Sudan. The mean counts of microfilariae from two skin snips, the number of microfilariae successfully sequenced and in the final dataset, and the number of haplotypes before and after treatment are given. The total number of parasite haplotypes per person are also indicated, as this number will be different from the sum of the pre-treatment and post-treatment haplotypes when haplotypes are found in both samples. Totals for each country represent the total number of unique pre- and post-treatment haplotypes used in the analysis; the total number in parentheses indicates the number of unique haplotypes across all worms, as some haplotypes were found in more than one person.

Country	Person	Mean Number of Microfilariae Pre/Post [37]	Number of Microfilariae in Dataset Pre/Post	Number of Haplotypes Pre/Post (Total)
DRC	OAE015	48.5/3	0/15	0/4 (4)
	OAE073	191/0	55/0	13/0 (13)
	OAE121	295.5/0	55/0	13/0 (13)
	OAE185	18.5/29	2/0	1/0 (1)
	OAE195	12.5/0	2/0	2/0 (2)
	OAE203	28/0	8/0	2/0 (2)
	OAE209	370.5/0	60/0	6/0 (6)
	OAE228	40/0	9/0	6/0 (6)
	OAE304	9/4	3/0	2/0 (2)
	OAE369	52.5/0	16/0	7/0 (7)
	All DRC		210/15	52/4 (52)
South Sudan	K014	16.5/20.5	0/1	0/1 (1)
	K028	78.5/20	27/0	16/0 (16)
	K029	57/143	0/1	0/1 (1)
	K038	23.5/24	3/0	3/0 (3)
	K096	108.5/1.5	9/0	8/0 (8)
	M204	59.5/46.5	27/10	13/8 (17)
	M206	105.5/62	33/21	19/ 16 (28)
	M219	45.5/64	13/0	8/0 (8)
	M224	23.5/51	28/9	15/7 (20)
	M238	23/55	23/6	14/5 (18)
	All South Sudan		163/48	96/38 (103)
Totals			373/63	148/42 (155)

**Figure 1:**
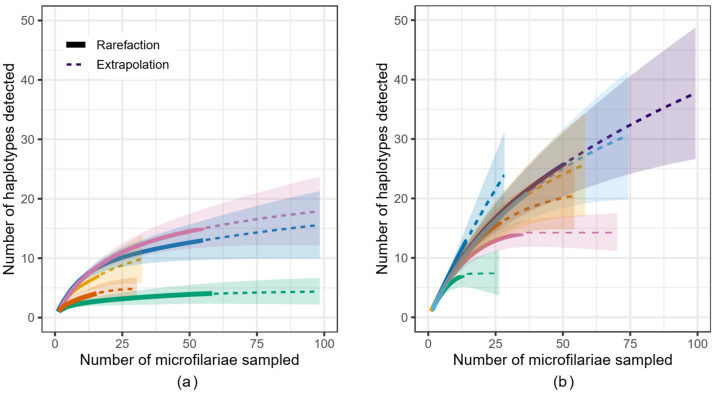
Rarefaction curve indicating how the number of *Onchocerca volvulus* microfilariae sampled from (**a**) five individual hosts from the DRC and (**b**) seven hosts from the South Sudan study areas affects the number of haplotypes likely to be observed. Solid line: rarefaction; dotted line: extrapolation; shaded area: confidence interval.

A rarefaction curve indicates how the number of haplotypes identified changes given the number of successfully sequenced microfilariae, and the shape of the curve indicates the extent to which the number of microfilariae sequenced effectively captures the genetic diversity within the population investigated, i.e., the microfilariae within an individual person (host). The rarefaction curves for samples from 12 individuals (those with microfilariae counts ≥10; Figure 1) suggest that, overall, the number of microfilariae successfully sequenced captured the genetic diversity in the parasite population in the DRC study area (green) to a larger extent than that in the South Sudan study area (purple). Extrapolated estimates for the total number of haplotypes predicted per person further suggest that the predicted number of reproductively active female worms (observed+unobserved) is greater in hosts from the South Sudan study area (average across people based on Chao estimate: 29.4 haplotypes per person; based on abundance-based accumulation: 36.4) when compared to the DRC (9.18 and 13.4, respectively), with highly variable associated standard errors (Table 2).

We used several different methods for assessing and comparing mtDNA genetic diversity in the microfilariae from the DRC and South Sudan locations. Because the sampling of the parasite population was non-random (i.e., the repeated sampling of sibling microfilariae in each host is non-representative of the population of parasites across all hosts), we estimated genetic diversity statistics using the set of unique maternal haplotypes rather than individual microfilariae sequences. Nucleotide diversity (π: the average number of pairwise nucleotide differences per site) of haplotypes from the DRC was 0.0198, and that of haplotypes from South Sudan was similar at 0.0212. Tajima’s D (the difference between diversity estimates derived from the mean number of pairwise differences and the number of variant sites) was negative, but not significantly so, in both populations (DRC: D = −2.591, *p* > 0.999; South Sudan: D = −2.609, *p* > 0.999). A simple, non-nested AMOVA calculated a fixation index of Φ_ST_ = 0.094 (*p* = 0.011) in the DRC and Φ_ST_ = 0.0351 (*p* = 0.1) in South Sudan. This indicates that in the DRC, most of this variation was partitioned within individual people (90.59%), with minor variation among people (9.41%). In South Sudan, this pattern of variation was even stronger (within individuals: 96.73%; between individuals 3.27%). In other words, the genetic differentiation between the microfilariae present in different people was marginally higher in the DRC than in South Sudan, but in both populations, nearly all of the genetic diversity was within the individual hosts (as in other parasites, such as the filarial nematode that causes lymphatic filariasis, *Wuchereria bancrofti* [77,78]).

**Figure 2:**
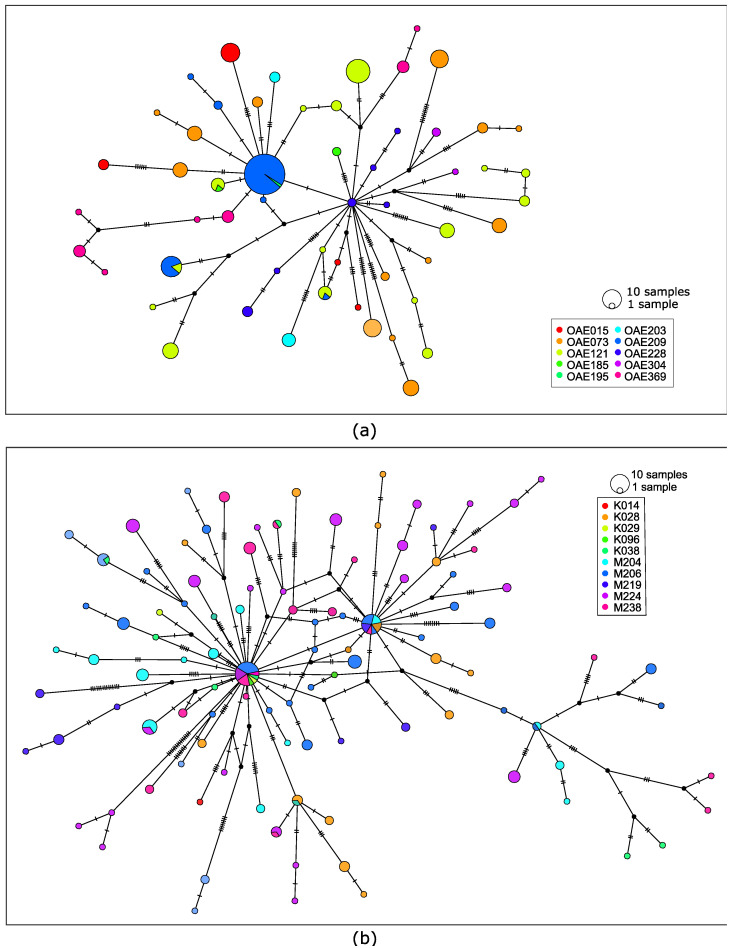
(**a**) Haplotype network using 143 mitochondrial single-nucleotide polymorphic variants from 225 microfilariae collected from 10 people from the DRC. (**b**) Haplotype network based on 228 genetic variants from 211 microfilariae collected from 10 people from South Sudan. Each circle represents a haplotype and is colored based on person; circle size indicates the number of microfilariae with that haplotype. Hatch marks along connecting lines indicate the number of sequence differences between haplotypes.

We visualized the relationships among the microfilariae using haplotype networks, which indicate how similar specific haplotypes are to each other. There are fewer haplotypes observed across the 225 microfilariae from 10 people from the DRC (Figure 2a) than observed across the 211 microfilariae from 10 people in South Sudan (Figure 2b), and the network is correspondingly simpler. There are two haplotypes that form central nodes in the South Sudan haplotype network that are shared by microfilariae from multiple people (Figure 2b). 

**Figure 3:**
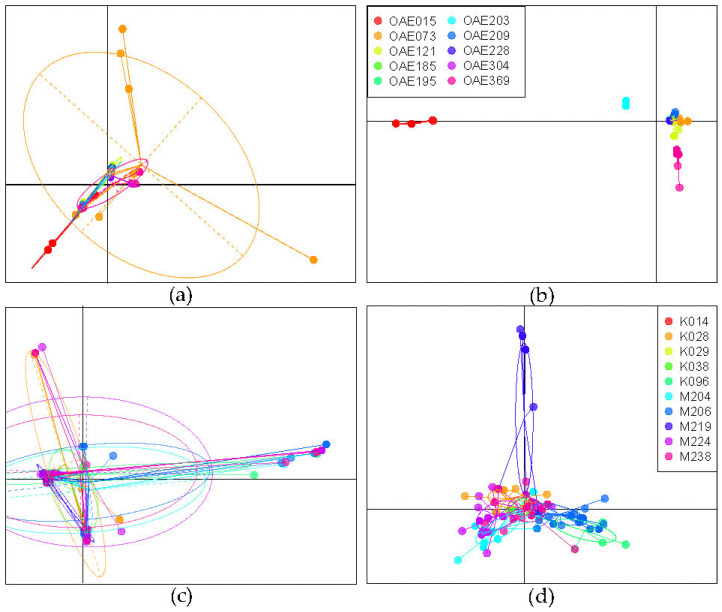
Analysis of genetic differentiation based on 320 mitochondrial DNA variant sites sequences of microfilariae collected from people in the DRC and South Sudan. (**a**) Principal components analysis (PCA) of microfilariae genotyped from 10 people from the DRC, colored by host (as in (**b**)); (**b**) discriminant analysis of principal components (DAPC) for microfilariae from DRC, maximizing differentiation between hosts; (**c**) PCA of microfilariae from 10 people from South Sudan, colored by host (as in (**d**)); (**d**) DAPC of microfilariae from South Sudan, maximizing differentiation between hosts.

We also explored whether genetic diversity was clustered by infrapopulation (the microfilariae within an individual host). The PCA of the samples from DRC suggests that there is considerable overlap in genetic diversity across microfilariae from different people (Figure 3a), but with only a few shared haplotypes (Figure 2a). DAPC is an analytical approach that maximizes differentiation among groups. A DAPC of the mitochondrial variants from DRC (using the first 80 PCs as determined by cross-validation) indicated that worms sampled from different people could be genetically differentiated (Figure 3b). While there are a few shared haplotypes, the proportion of individual microfilariae that could be correctly assigned to their host, based on their mitochondrial genotype, is 0.8756. The assignment proportion does not necessarily serve as a metric for similarity among worms *within* a person; rather, it indicates how different the worms sampled are *between* people. The higher the assignment proportion, the more distinct the parasite genotypes present in different people.

The PCA of mitochondrial genetic variation in the South Sudan microfilariae similarly suggests overlap in genetic diversity across the microfilariae in different hosts (Figure 3c), with a couple of haplotypes found in several people (Figure 2b). DAPC (using the first 60 PCs) was less able to discriminate among the parasites from different people compared to the DAPC performed on the DRC samples (Figure 3d), and the assignment proportion for individual microfilariae to the infected person from which they came was only 0.641, indicating that discriminating infrapopulations based on maternal mitochondrial haplotype was not feasible.

**Figure 4:**
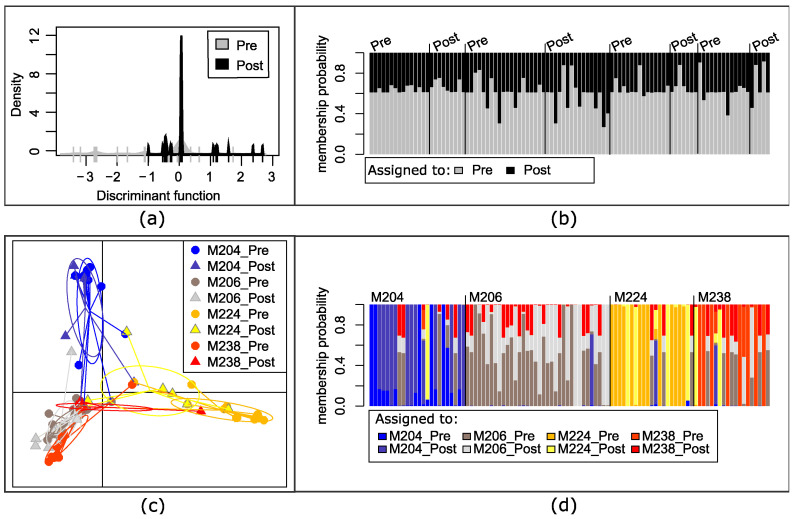
Analysis of genetic differentiation based on the mitochondrial genotypes of microfilariae collected from four people in South Sudan with both pre- and post-treatment samples. (**a**) Discriminate analysis of principal components (DAPC) by time collected: pre-treatment (pre) or 5 months post-treatment (post). (**b**) Each column represents a mitochondrial haplotype found within a person and the shading the probability that haplotype would be assigned to time of collection based on the DAPC, with the actual day collected indicated above and columns arranged by individual person, as in (**d**); (**c**) DAPC of haplotypes maximizing differentiation between each individual host and by day collected; (**d**) each column represents a haplotype within a person (M204, M206, M224, or M238) and the shading the probability that it would be assigned to the pre- or post-treatment sample of one of the four individuals.

We focused further analysis on the 98 microfilarial haplotypes in the four people from South Sudan from whom we had both pre- and post-ivermectin treatment samples. It was not possible to discriminate haplotypes based on whether they were collected prior to or following treatment: haplotypes found before treatment (gray) were also found post-treatment (black) and some of the haplotypes were found in more than one person. Figure 4a visually demonstrates this genetic overlap between microfilariae haplotypes, and Figure 4b indicates the inability of the discriminant function to differentiate between pre- and post-treatment haplotypes: each haplotype identified (columns) has a similar probability of being assigned to either the pre-treatment (gray) or post-treatment (black) category based on their genotype. When discriminating based on human host *and* the day sampled, the post-treatment haplotypes largely overlapped with the pre-treatment haplotypes sampled from the same individual, although haplotypes from different individuals were moderately differentiated (Figure 4c,d).

### 3.3. Genetic Variation in the African Context

Maternal haplotype sequences from the DRC and South Sudan mf data (i.e., retaining only one copy of a haplotype if it was found in multiple people and/or at multiple time points) were combined with sequences from Guinea (*n* = 4), Sierra Leone (*n* = 3), Liberia (*n* = 1), Mali (*n* = 10), Côte d’Ivoire (*n* = 13), Ghana (*n* = 189), Benin (*n* = 1), Cameroon (*n* = 39), and Uganda (*n* = 2) [49,50] for a total of 438 *O. volvulus*. After filtering, there were 851 single-nucleotide polymorphic variants called in total; of these, 427 were singletons. For downstream analyses, the singletons were removed.

Of the 424 polymorphic variants in the 13,744 bp mitochondrial genome, 139 variants were nonsynonymous (i.e., resulting in a change in the amino acid in the gene product), 208 were synonymous (i.e., a nonsynonymous:synonymous ratio across all genes of 0.668), 4 were nonsense mutations that would truncate the predicted gene product, and the remaining were in non-coding regions. The overall nucleotide diversity was 0.0111. The reduction in nucleotide diversity compared to that estimated from microfilariae from the DRC and South Sudan was largely driven by low nucleotide diversity among the 221 West African worms (π = 0.0064). Most of the variance in this diversity was within countries rather than between them: a simple non-nested AMOVA resulted in a fixation index of Φ_ST_ = 0.0255 (*p* = 0.009; significance based on 1000 permutations), where only 2.55% variance was among countries and 97.45% was within country.

**Figure 5:**
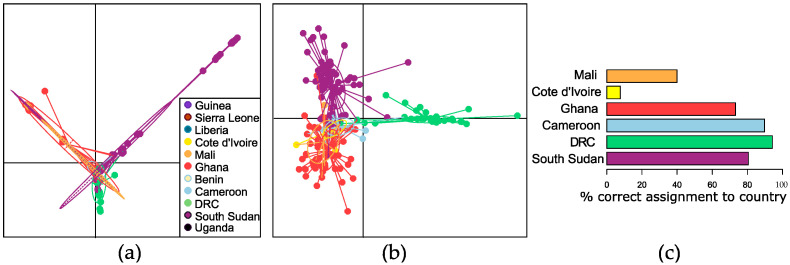
(**a**) Principal component analysis of *Onchocerca volvulus* worms from Benin, Cameroon, Côte d’Ivoire, Democratic Republic of Congo (DRC), Ghana, Guinea, Liberia, Mali, Sierra Leone, South Sudan, and Uganda based on mitochondrial genome sequencing. (**b**) Discriminant analysis of principal components (DAPC) of worms from Cameroon, Côte d’Ivoire, DRC, Mali, and South Sudan. (**c**) Percentage of worms from each country that were correctly assigned to their country of origin based on DAPC.

Based on PCA, the haplotype diversity observed in South Sudan and DRC contained some sequence variation that overlapped with mitochondrial sequences obtained from adult female worms from West and Central Africa, and some sequence variation that was quite distinct (Figure 5a; consistent with the haplotype network presented in Figure S1). The results of a DAPC including only parasites from those countries where at least 10 parasites were sampled (Cameroon, Côte d’Ivoire, DRC, Ghana, Mali, and South Sudan), using the first 70 PCAs as determined through cross-validation, was visually consistent with the results of the PCA (Figure 5b). Assignment proportions (the proportion of parasites correctly assigned to their country of origin) were higher for parasites from Cameroon (0.90), DRC (0.94), and South Sudan (0.81) than parasites from Ghana (0.73), Côte d’Ivoire (0.08), and Mali (0.40) (Figure 5c). The very low proportions for Côte d’Ivoire and Mali could be due to fewer haplotypes being available compared to the number of sequences from countries with higher assignment proportions (i.e., an analytical bias). Consideration has to be given to the possibility that parasites in countries in West Africa are more closely related overall (due to the migration of hosts or vectors and/or the more recent divergence of those populations in evolutionary history) and thus less able to be discriminated using mitochondrial data alone.

## 4. Discussion

Our results showed that the *O. volvulus* mitochondrial DNA haplotype diversity varied between people and that the number of haplotypes in a single person was in the range of 2–26. The number of haplotypes indicates the minimum number of adult female worms that produced the microfilariae in our analyses, and is within the range of previous estimates of the number of female adult worms per person in West African populations [35,36]. Furthermore, our analyses showed that: (1) the number of haplotypes identified increased with the number of microfilariae successfully sequenced (Figure 1), indicating that data from a higher number of microfilariae than was available to us is required to estimate the number of haplotypes within one host as well as within the human population to which the host belongs; (2) the total number of microfilariae that need to be successfully sequenced to capture the genomic diversity of their parents will vary depending on the number of adult parasites within a person and geographic location (Figure 1, Table 2); and, thus, that (3) the number of haplotypes we identified underestimates the number of reproductively active female worms in most people, more so in those from South Sudan than those from DRC. If a rarefaction curve begins to asymptote, then the microfilariae genotyped is more likely to be representative of the genetic diversity within that person. In South Sudan, the number of microfilariae per person that would need to be successfully sequenced to capture the genetic diversity in that parasite population appears to be higher than for people in the DRC (Figure 1). These differences were observed despite similar numbers of microfilariae sequenced from a similar number of hosts, which further suggests that the intensity of infection, or worm burden per person, is higher in the area sampled in South Sudan than in DRC. The differences in infection intensity may be related to differences in prevalence; in the cohorts studied here, 36.5% of people recruited for the study based in Ituri compared to 84.9% of participants from South Sudan were microfilaridermic [38,40]. However, because the participants did not represent a random sampling of onchocerciasis-infected people in either location, sampling parasites from additional people would be required to confirm what would be a significant epidemiological difference between the two areas. Differences in onchocerciasis prevalence are driven by differences in annual biting rates, which are, in turn, driven by ecological conditions favorable for blackfly breeding, the amount of time people spend in areas with different biting rates, and the presence and effectiveness of interventions [79,80,81].

The statistically significant fixation index (Φ_ST_ from AMOVA) of haplotypes from the DRC and South Sudan demonstrates that each individual infected person carries a genetically variable population of worms. In population genetic terms, each infected person carries their own infrapopulation that is drawn from a much larger pool of parasites present in the population as a whole (the metapopulation). Most of the genetic variation is partitioned within people, which means that the differentiation between infrapopulations is moderate, and stronger in the DRC than South Sudan. The difference between the DRC and South Sudan infrapopulation differentiation is driven by fewer haplotypes (i.e., fewer reproductively active females) detected per host from the DRC, and thus that each sampled host contained a smaller proportion of the total parasite population’s genetic diversity than hosts in South Sudan. Regardless, the higher within- than between-infrapopulation diversity supports the hypothesis that the parasite populations in each transmission zone—the geographic area over which parasites are transmitted and thus able to interbreed—are large, and the probability of parasites that have identical-by-descent mitochondrial haplotypes being transmitted to one person is low. 

The *O. volvulus* populations sampled in DRC and South Sudan harbor population-associated genetic variants that can be used to discriminate many of the parasites collected from these locations from parasites found elsewhere in sub-Saharan Africa. As shown in similar analyses of mitochondrial DNA data from *O. volvulus* [49,78], there is a widespread haplotype found in nearly all countries, which causes the overlap in genetic diversity observed (Figure 5; central node in Figure S1). This is consistent with large population sizes maintaining ancestral variation in mitochondrial genomes, which are under selective constraint to retain function.

For the four hosts from South Sudan from whom we had mitochondrial DNA sequences from microfilariae collected before as well as five months post-ivermectin treatment, we found that the haplotypes of the microfilariae collected post treatment were not distinct from those taken before treatment (Figure 4b). This shared genetic diversity indicates that the microfilariae present before and five months after ivermectin treatment are likely the offspring of those mothers that recovered fertility relatively rapidly. However, some haplotypes identified post treatment were not found in the pre-treatment sample. New post-treatment infections can be discounted as a source of microfilariae in the skin only five months post treatment, given that it takes 12–18 months for L3 larvae transmitted to mature into a reproductive adult worm and that it might take up to 3 years for the microfilariae of that adult to be sufficiently numerous to be detectable in skin snips [32,82,83,84]. An alternative source for post-treatment mitochondrial haplotypes not detected in a pre-treatment sample could be the random assortment of unique haplotypes into offspring from a heteroplasmic mother (i.e., a female worm with more than one mitochondrial haplotype). A likely interpretation is that sampling was not sufficient to detect all of the genetic diversity actually present within the host: some genotypes are missing from the pre-treatment sample because of under-sampling rather than true absence. We tested this hypothesis by producing a rarefaction curve, which indicates how the detection of unique mitochondrial sequences (or haplotypes) found in each person in both the DRC and South Sudan microfilariae increases with additional sequenced microfilariae (Figure 1). Under the assumption that post-treatment microfilariae would be a genetic subset of the pre-treatment worms, the results are consistent with the hypothesis that there was insufficient sampling of microfilariae from people in the South Sudan cohort to capture the genetic diversity present at the time of ivermectin treatment. Extrapolation may be useful for guiding researchers in determining when additional sequencing is required; while the extrapolated number of haplotypes varies depending on the assumptions made about detection of rare haplotypes [70,71], we found that in as many as three people (M206, M224, M238), only about half of the mitochondrial diversity was predicted to have been sampled (Table 2).

The analysis of parasite mitochondrial data to track changes in the number of reproductively active female worms could be useful for elimination programs and researchers working where there has been persistent transmission despite MDAi with high compliance and community coverage. High vector-biting rates can sustain transmission, even when skin microfilariae load is low. However, ongoing transmission might also be driven by parasites transmitted via vector or human movement from other onchocerciasis endemic areas or by SOR to ivermectin (reviewed in [85]). Changes in the estimated number of reproductively active worms over time could serve as an indicator of whether years of programmatic MDAi are successfully reducing the worm burden, or whether new imported infections (represented as new haplotypes) are identified. In this latter case, the genotyping of worms from areas with ongoing transmission that are likely to be connected by movement of people (as in [86,87]) or vectors (including long-distance, wind-assisted migration [88,89,90,91]) could help determine whether transmission among endemic areas might be contributing to persistent prevalence (see, e.g., [92,93] for modeling that explores the impact of movement of people or vectors on prevalence). We have shown here that mitochondrial haplotypes can discriminate among parasites from different countries, and, thus, may be informative where cross-border transmission might occur. Nuclear genotypes derived from the whole-genome sequencing of adult worms were able to discriminate *O. volvulus* collected in forest vs. savannah bioclimes within West Africa [49]; thus, the further development of cost-effective approaches for the nuclear genotyping of microfilariae would be useful for identifying transmission occurring between endemic areas.

In areas where SOR is suspected or has been demonstrated, monitoring changes in the number of reproductive SOR macrofilariae can help inform decisions about whether to deploy certain alternative and/or complementary interventions. SOR macrofilariae resume reproduction as soon as a few weeks after ivermectin treatment. Assuming there is no difference in the growth, survival, and probability of transmission of microfilariae that are the offspring of SOR and non-SOR macrofilariae, and assuming that the early resumption of reproduction is a heritable trait [20], the prevalence of reproducing SOR parasites will increase over time. This would jeopardize onchocerciasis control and elimination efforts and would require alternative intervention strategies. To date, the methods for identifying SOR parasites involve counting the microfilariae in skin snips and/or evaluating developmental stages in the uteri of macrofilariae excised from palpable subcutaneous nodules (where a fraction of the macrofilariae reside) soon (typically around 3 months) after ivermectin treatment [16,17,18,19,20,21,22,23,24,26]. Neither method can identify the number or percentage of SOR macrofilariae. A genetic approach applied to longitudinal, post-treatment samples of skin microfilariae or parasites in the vectors could indicate whether or not there is an increase in the number of SOR adult female worms producing those microfilariae repopulating the skin early, and, thus, whether alternative intervention strategies should be considered. 

A major challenge for developing a genetics-based tool is the expense and sampling effort required to sufficiently capture the genetic diversity of reproducing parasites. A cost-effective approach would minimize the amount of effort needed to obtain microfilariae from skin snips, be able to sequence DNA from parasite pools, and ideally be suitable for use with blackfly pools. Long-read sequencing technologies (such as Oxford Nanopore) applied to amplified targets in the mitochondrial or nuclear genome would have the advantage of sequencing haplotypes even from DNA pools. Since national NTD programs are currently not set up for sequencing and population genetic analysis, collaboration with a research institution in the country, or building the required infrastructure and personnel capacity within the programs, would be needed.

Finally, the methods we present here may be useful, and easiest to implement, in studies evaluating the efficacy of new drugs, when it is important to know whether microfilariae appearing in the skin are due to macrofilariae reproductively active before treatment and that were not sterilized or killed by the new drug or whether they are due to new infections acquired after treatment [94]. In drug efficacy trials, four skin snips are usually taken before treatment and at varying time points after treatment. The mitochondrial haplotypes of the microfilariae sampled before and after treatment (timing dependent on the prior knowledge about the effect of the drug on the parasites) can be compared to estimate the effect of a drug on the burden of reproductively active female worms and on individual female worm fertility. Depending on the extent to which sampling captures haplotype diversity, samples taken >1 year after treatment could provide insight into the probability that the haplotypes only identified in post-treatment samples are due to new infections rather than due to parasites not affected by or having recovered from the effect of the drug. This is particularly important when the studies are conducted in areas with high transmission that could result in post-treatment infection.

## Figures and Tables

**Table 2:** Extrapolated mitochondrial haplotype richness estimates with standard error (SE) for *Onchocerca volvulus* microfilariae sampled from participants in the DRC and South Sudan. Table indicating the sample size (number of microfilariae sequenced), observed number of haplotypes, extrapolated number of haplotypes based on the Chao [69] and the abundance-based coverage estimator (ACE), adjusted for sample bias [70,71].

Person	Sample Size	Observed No. Haplotypes	Chao 1 (SE)	ACE (SE)
DRC: OAE015	15	4	5.0 (2.17)	16.0 (4.14)
DRC: OAE073	55	13	16.0 (4.14)	15.61 (1.47)
DRC: OAE121	55	15	18.3 (4.10)	18.66 (1.89)
DRC: OAE209	58	4	4.0 (0.22)	5.24 (1.31)
DRC: OAE369	16	7	13.0 (7.08)	11.47 (1.49)
South Sudan: K028	27	16	20.7 (4.48)	22.74 (2.33)
South Sudan: K096	14	13	46.0 (26.3)	91.0 (0.98)
South Sudan: M204	35	14	14.1 (0.49)	14.85 (1.19)
South Sudan: M206	51	26	52.3 (18.74)	45.66 (3.50)
South Sudan: M219	13	7	7.2 (0.62)	8.27 (1.36)
South Sudan: M224	37	21	36.6 (11.63)	40.59 (3.18)
South Sudan: M238	29	18	29.0 (8.86)	31.53 (2.69)

## Data Availability

The data presented in this study are openly available from the National Center for Biotechnology Information Short Read Archive (https://www.ncbi.nlm.nih.gov/sra/) under study accession number PRJNA981587.

## Supplementary Materials

The following supporting information can be downloaded at: https://www.mdpi.com/article/10.3390/pathogens12070971/s1, Figure S1: Haplotype network of mitochondrial haplotypes of *Onchocerca volvulus* collected in Benin, Cameroon, Côte d’Ivoire, Democratic Republic of Congo, Ghana, Guinea, Liberia, Mali, Sierra Leone, South Sudan, and Uganda; Table S1: Sequencing and mapping statistics for *Onchocerca volvulus* collected in sub-Saharan Africa.

## References

[1] World Health Organization (2021). Elimination of human onchocerciasis: Progress report, 2020. Wkly. Epidemiol. Rec..

[2] Chesnais C.B., Nana-Djeunga H.C., Njamnshi A.K., Lenou-Nanga C.G., Boulle C., Bissek A.Z., Kamgno J., Colebunders R., Boussinesq M. (2018). The temporal relationship between onchocerciasis and epilepsy: A population-based cohort study. Lancet Infect. Dis..

[3] Colebunders R., Njamnshi A.K., van Oijen M., Mukendi D., Kashama J.M., Mandro M., Gumisiriza N., Preux P.M., Suykerbuyk P., Idro R. (2017). Onchocerciasis-associated epilepsy: From recent epidemiological and clinical findings to policy implications. Epilepsia Open.

[4] Colebunders R., Njamnshi A.K., Menon S., Newton C.R., Hotterbeekx A., Preux P.M., Hopkins A., Vaillant M., Siewe Fodjo J.N. (2021). *Onchocerca volvulus* and epilepsy: A comprehensive review using the Bradford Hill criteria for causation. PLoS Negl. Trop. Dis..

[5] World Health Organization (2020). Ending the Neglect to Attain the Sustainable Development Goals: A Road Map for Neglected Tropical Diseases 2021–2030.

[6] Plaisier A.P., Alley E.S., Boatin B.A., Van Oortmarssen G.J., Remme H., De Vlas S.J., Bonneux L., Habbema J.D. (1995). Irreversible effects of ivermectin on adult parasites in onchocerciasis patients in the Onchocerciasis Control Programme in West Africa. J. Infect. Dis..

[7] Basáñez M.-G., Pion S.D., Boakes E., Filipe J.A., Churcher T.S., Boussinesq M. (2008). Effect of single-dose ivermectin on *Onchocerca volvulus*: A systematic review and meta-analysis. Lancet Infect. Dis..

[8] Bottomley C., Isham V., Collins R.C., Basáñez M.G. (2008). Rates of microfilarial production by *Onchocerca volvulus* are not cumulatively reduced by multiple ivermectin treatments. Parasitology.

[9] Turner H.C., Walker M., Churcher T.S., Basáñez M.G. (2014). Modelling the impact of ivermectin on River blindness and its burden of morbidity and mortality in African savannah: EpiOncho projections. Parasit. Vectors.

[10] Sauerbrey M., Rakers L.J., Richards F.O. (2018). Progress toward elimination of onchocerciasis in the Americas. Int. Health.

[11] World Health Organization (2022). Elimination of human onchocerciasis: Progress report, 2021—Élimination de l’onchocercose humaine: Rapport de situation, 2021. Wkly. Epidemiol. Rec..

[12] Tekle A.H., Zouré H.G., Noma M., Boussinesq M., Coffeng L.E., Stolk W.A., Remme J.H. (2016). Progress towards onchocerciasis elimination in the participating countries of the African Programme for Onchocerciasis Control: Epidemiological evaluation results. Infect. Dis. Poverty.

[13] Diawara L., Traore M.O., Badji A., Bissan Y., Doumbia K., Goita S.F., Konate L., Mounkoro K., Sarr M.D., Seck A.F. (2009). Feasibility of onchocerciasis elimination with ivermectin treatment in endemic foci in Africa: First evidence from studies in Mali and Senegal. PLoS Negl. Trop. Dis..

[14] Traore M.O., Sarr M.D., Badji A., Bissan Y., Diawara L., Doumbia K., Goita S.F., Konate L., Mounkoro K., Seck A.F. (2012). Proof-of-principle of onchocerciasis elimination with ivermectin treatment in endemic foci in Africa: Final results of a study in Mali and Senegal. PLoS Negl. Trop. Dis..

[15] World Health Organization, African Programme for Onchocerciasis Control (2015). Report of the Consultative Meetings on Strategic Options and Alternative Treatment Strategies for Accelerating Onchocerciasis Elimination in Africa.

[16] Awadzi K., Attah S.K., Addy E.T., Opoku N.O., Quartey B.T., Lazdins-Helds J.K., Ahmed K., Boatin B.A., Boakye D.A., Edwards G. (2004). Thirty-month follow-up of sub-optimal responders to multiple treatments with ivermectin, in two onchocerciasis-endemic foci in Ghana. Ann. Trop. Med. Parasitol..

[17] Awadzi K., Boakye D.A., Edwards G., Opoku N.O., Attah S.K., Osei-Atweneboana M.Y., Lazdins-Helds J.K., Ardrey A.E., Addy E.T., Quartey B.T. (2004). An investigation of persistent microfilaridermias despite multiple treatments with ivermectin, in two onchocerciasis-endemic foci in Ghana. Ann. Trop. Med. Parasitol..

[18] Churcher T.S., Pion S.D., Osei-Atweneboana M.Y., Prichard R.K., Awadzi K., Boussinesq M., Collins R.C., Whitworth J.A., Basáñez M.G. (2009). Identifying sub-optimal responses to ivermectin in the treatment of River Blindness. Proc. Natl. Acad. Sci. USA.

[19] Osei-Atweneboana M.Y., Eng J.K., Boakye D.A., Gyapong J.O., Prichard R.K. (2007). Prevalence and intensity of *Onchocerca volvulus* infection and efficacy of ivermectin in endemic communities in Ghana: A two-phase epidemiological study. Lancet.

[20] Doyle S.R., Bourguinat C., Nana-Djeunga H.C., Kengne-Ouafo J.A., Pion S.D.S., Bopda J., Kamgno J., Wanji S., Che H., Kuesel A.C. (2017). Genome-wide analysis of ivermectin response by *Onchocerca volvulus* reveals that genetic drift and soft selective sweeps contribute to loss of drug sensitivity. PLoS Negl. Trop. Dis..

[21] Osei-Atweneboana M.Y., Awadzi K., Attah S.K., Boakye D.A., Gyapong J.O., Prichard R.K. (2011). Phenotypic evidence of emerging ivermectin resistance in *Onchocerca volvulus*. PLoS Negl. Trop. Dis..

[22] Nana-Djeunga H.C., Bourguinat C., Pion S.D., Bopda J., Kengne-Ouafo J.A., Njiokou F., Prichard R.K., Wanji S., Kamgno J., Boussinesq M. (2014). Reproductive status of *Onchocerca volvulus* after ivermectin treatment in an ivermectin-naive and a frequently treated population from Cameroon. PLoS Negl. Trop. Dis..

[23] Opoku N.O., Bakajika D.K., Kanza E.M., Howard H., Mambandu G.L., Nyathirombo A., Nigo M.M., Kasonia K., Masembe S.L., Mumbere M. (2018). Single dose moxidectin versus ivermectin for *Onchocerca volvulus* infection in Ghana, Liberia, and the Democratic Republic of the Congo: A randomised, controlled, double-blind phase 3 trial. Lancet.

[24] Bakajika D., Kanza E.M., Opoku N.O., Howard H.M., Mambandu G.L., Nyathirombo A., Nigo M.M., Kennedy K.K., Masembe S.L., Mumbere M. (2022). Effect of a single dose of 8 mg moxidectin or 150 μg/kg ivermectin on *O. volvulus* skin microfilariae in a randomized trial: Differences between areas in the Democratic Republic of the Congo, Liberia and Ghana and impact of intensity of infection. PLoS Negl. Trop. Dis..

[25] Awadzi K., Opoku N.O., Attah S.K., Lazdins-Helds J., Kuesel A.C. (2014). A randomized, single-ascending-dose, ivermectin-controlled, double-blind study of moxidectin in *Onchocerca volvulus* infection. PLoS Negl. Trop. Dis..

[26] Frempong K.K., Walker M., Cheke R.A., Tetevi E.J., Gyan E.T., Owusu E.O., Wilson M.D., Boakye D.A., Taylor M.J., Biritwum N.K. (2016). Does increasing treatment frequency address suboptimal responses to ivermectin for the control and elimination of River blindness?. Clin. Infect. Dis..

[27] Abong R.A., Amambo G.N., Chounna Ndongmo P.W., Njouendou A.J., Ritter M., Beng A.A., Esum M.E., Deribe K., Fru-Cho J., Fombad F.F. (2020). Differential susceptibility of *Onchocerca volvulus* microfilaria to ivermectin in two areas of contrasting history of mass drug administration in Cameroon: Relevance of microscopy and molecular techniques for the monitoring of skin microfilarial repopulation within six months of direct observed treatment. BMC Infect. Dis..

[28] Stolk W.A., Walker M., Coffeng L.E., Basáñez M.G., de Vlas S.J. (2015). Required duration of mass ivermectin treatment for onchocerciasis elimination in Africa: A comparative modelling analysis. Parasit. Vectors.

[29] Basáñez M.G., Walker M., Turner H.C., Coffeng L.E., de Vlas S.J., Stolk W.A. (2016). River blindness: Mathematical models for control and elimination. Adv. Parasitol..

[30] Duke B.O. (1970). Onchocerciasis; deep worm bundles close to hip joints. Trans. R. Soc. Trop. Med. Hyg..

[31] Meyers W.M., Neafie R.C., Connor D.H. (1977). Onchocerciasis: Invasion of deep organs by *Onchocerca volvulus*. Am. J. Trop. Med. Hyg..

[32] Duke B.O. (1993). The population dynamics of *Onchocerca volvulus* in the human host. Trop. Med. Parasitol..

[33] Duerr H.P., Dietz K., Schulz-Key H., Buttner D.W., Eichner M. (2004). The relationships between the burden of adult parasites, host age and the microfilarial density in human onchocerciasis. Int. J. Parasitol..

[34] Albiez E.J. (1983). Studies on nodules and adult *Onchocerca volvulus* during a nodulectomy trial in hyperendemic villages in Liberia and Upper Volta. I. Palpable and impalpable onchocercomata. Tropenmed. Parasitol..

[35] Basáñez M.G., Boussinesq M. (1999). Population biology of human onchocerciasis. Philos. Trans. R. Soc. Lond. B Biol. Sci..

[36] Schulz-Key H., Albiez E.J. (1977). Worm burden of *Onchocerca volvulus* in a hyperendemic village of the rain-forest in West Africa. Tropenmedizin Parasitol..

[37] Dusabimana A., Bhwana D., Raimon S., Mmbando B.P., Hotterbeekx A., Tepage F., Mandro M., Siewe Fodjo J.N., Abrams S., Colebunders R. (2020). Ivermectin treatment response in *Onchocerca volvulus* infected persons with epilepsy: A three-country short cohort study. Pathogens.

[38] Fodjo J.N.S., Mandro M., Mukendi D., Tepage F., Menon S., Nakato S., Nyisi F., Abhafule G., Wonya’rossi D., Anyolito A. (2019). Onchocerciasis-associated epilepsy in the Democratic Republic of Congo: Clinical description and relationship with microfilarial density. PLoS Negl. Trop. Dis..

[39] Mandro M., Siewe Fodjo J.N., Dusabimana A., Mukendi D., Haesendonckx S., Lokonda R., Nakato S., Nyisi F., Abhafule G., Wonya’rossi D. (2020). Single versus multiple dose ivermectin regimen in onchocerciasis-infected persons with epilepsy treated with phenobarbital: A randomized clinical trial in the Democratic Republic of Congo. Pathogens.

[40] Abd-Elfarag G., Carter J.Y., Raimon S., Sebit W., Suliman A., Fodjo J.N.S., Olore P.C., Biel K.P., Ojok M., Logora M.Y. (2020). Persons with onchocerciasis-associated epilepsy and nodding seizures have a more severe form of epilepsy with more cognitive impairment and higher levels of *Onchocerca volvulus* infection. Epileptic Disord..

[41] Colebunders R., Carter J.Y., Olore P.C., Puok K., Bhattacharyya S., Menon S., Abd-Elfarag G., Ojok M., Ensoy-Musoro C., Lako R. (2018). High prevalence of onchocerciasis-associated epilepsy in villages in Maridi County, Republic of South Sudan: A community-based survey. Seizure.

[42] Post R.J., Laudisoit A., Mandro M., Lakwo T., Laemmer C., Pfarr K., Hoerauf A., Tortosa P., Gomard Y., Ukety T. (2022). Identification of the onchocerciasis vector in the Kakoi-Koda focus of the Democratic Republic of Congo. PLoS Negl. Trop. Dis..

[43] Nana-Djeunga H.C., Sicard C.M., Mogoung-Wafo A.E., Chesnais C.B., Deleglise H., Touka-Nounkeu R., Domche A., Golden A., Klion A.D., Nutman T.B. (2022). Changes in onchocerciasis Ov16 IgG4 rapid diagnostic test results over one-month follow-up: Lessons for reading timeframe and decision-making. Am. J. Trop. Med. Hyg..

[44] Hotterbeekx A., Perneel J., Mandro M., Abhafule G., Siewe Fodjo J.N., Dusabimana A., Abrams S., Kumar-Singh S., Colebunders R. (2020). Comparison of diagnostic tests for *Onchocerca volvulus* in the Democratic Republic of Congo. Pathogens.

[45] World Health Organization (2023). Report of the Proceedings of the Fifth Meeting of the Onchocerciasis Technical Advisory Subgroup, Virtual Meeting, 9–10 December 2021.

[46] World Health Organization (2021). Report on the Fourth Meeting of the WHO Onchocerciasis Technical Advisory Subgroup: Virtual Meeting, 28–29 October 2020.

[47] Dusabimana A., Siewe Fodjo J.N., Ndahura M.M., Mmbando B.P., Jada S.R., Boven A., De Smet E., Ukety T., Njamnshi A.K., Laudisoit A. (2022). Surveillance for onchocerciasis-associated epilepsy and OV16 IgG4 testing of children 6–10 years old should be used to identify areas where onchocerciasis elimination programs need strengthening. Pathogens.

[48] Lakwo T.L., Raimon S., Tionga M., Siewe Fodjo J.N., Alinda P., Sebit W.J., Carter J.Y., Colebunders R. (2020). The role of the Maridi Dam in causing an onchocerciasis-associated epilepsy epidemic in Maridi, South Sudan: An epidemiological, sociological, and entomological study. Pathogens.

[49] Choi Y.J., Tyagi R., McNulty S.N., Rosa B.A., Ozersky P., Martin J., Hallsworth-Pepin K., Unnasch T.R., Norice C.T., Nutman T.B. (2016). Genomic diversity in *Onchocerca volvulus* and its *Wolbachia* endosymbiont. Nat. Microbiol..

[50] Crawford K.E., Hedtke S.M., Kuesel A.C., Doyle S.D., Armoo S., Osei-Atweneboana M., Grant W.N. (2019). Genome-based tools for onchocerciasis elimination: Utility of the mitochondrial genome for delineating *Onchocerca volvulus* transmission zones. bioRxiv.

[51] Bustin S.A., Benes V., Garson J.A., Hellemans J., Huggett J., Kubista M., Mueller R., Nolan T., Pfaffl M.W., Shipley G.L. (2009). The MIQE guidelines: Minimum information for publication of quantitative real-time PCR experiments. Clin. Chem..

[52] Burns M., Valdivia H. (2008). Modelling the limit of detection in real-time quantitative PCR. Eur. Food Res. Technol..

[53] Bolger A.M., Lohse M., Usadel B. (2014). Trimmomatic: A flexible trimmer for Illumina sequence data. Bioinformatics.

[54] Cotton J.A., Bennuru S., Grote A., Harsha B., Tracey A., Beech R., Doyle S.R., Dunn M., Hotopp J.C., Holroyd N. (2016). The genome of *Onchocerca volvulus*, agent of river blindness. Nat. Microbiol..

[55] Li H. (2013). Aligning sequence reads, clone sequences and assembly contigs with BWA-MEM. arXiv.

[56] Li H., Durbin R. (2009). Fast and accurate short read alignment with Burrows-Wheeler transform. Bioinformatics.

[57] Li H., Handsaker B., Wysoker A., Fennell T., Ruan J., Homer N., Marth G., Abecasis G., Durbin R., 1000 Genome Project Data Processing Subgroup (2009). The Sequence Alignment/Map format and SAMtools. Bioinformatics.

[58] Quinlan A.R. (2014). BEDTools: The Swiss-Army Tool for Genome Feature Analysis. Curr. Protoc. Bioinforma..

[59] Price A.L., Jones N.C., Pevzner P.A. (2005). De novo identification of repeat families in large genomes. Bioinformatics.

[60] Garrison E., Marth G. (2012). Haplotype-based variant detection from short-read sequencing. arXiv.

[61] Danecek P., Auton A., Abecasis G., Albers C.A., Banks E., DePristo M.A., Handsaker R.E., Lunter G., Marth G.T., Sherry S.T. (2011). The variant call format and VCFtools. Bioinformatics.

[62] Danecek P., Bonfield J.K., Liddle J., Marshall J., Ohan V., Pollard M.O., Whitwham A., Keane T., McCarthy S.A., Davies R.M. (2021). Twelve years of SAMtools and BCFtools. Gigascience.

[63] Garrison E., Kronenberg Z.N., Dawson E.T., Pedersen B.S., Prins P. (2021). Vcflib and tools for processing the VCF variant call format. bioRxiv.

[64] Jombart T., Ahmed I. (2011). adegenet 1.3-1: New tools for the analysis of genome-wide SNP data. Bioinformatics.

[65] (2008). R Development Core Team R: A Language and Environment for Statistical Computing.

[66] Jombart T., Devillard S., Balloux F. (2010). Discriminant analysis of principal components: A new method for the analysis of genetically structured populations. BMC Genet..

[67] Hsieh T.C., Ma K.H., Chao A. (2016). iNEXT: An R package for rarefaction and extrapolation of species diversity (Hill numbers). Methods Ecol. Evol..

[68] Dixon P. (2003). VEGAN, a package of R functions for community ecology. J. Veg. Sci..

[69] Chao A. (1987). Estimating the population size for capture-recapture data with unequal catchability. Biometrics.

[70] Chiu C.H., Wang Y.T., Walther B.A., Chao A. (2014). An improved nonparametric lower bound of species richness via a modified good-turing frequency formula. Biometrics.

[71] O’Hara R.B. (2005). Species richness estimators: How many species can dance on the head of a pin?. J. Anim. Ecol..

[72] Cingolani P., Platts A., Wang L.L., Coon M., Nguyen T., Wang L., Land S.J., Lu X., Ruden D.M. (2012). A program for annotating and predicting the effects of single nucleotide polymorphisms, SnpEff: SNPs in the genome of *Drosophila melanogaster* strain w1118; iso-2; iso-3. Fly.

[73] Lischer H.E., Excoffier L. (2012). PGDSpider: An automated data conversion tool for connecting population genetics and genomics programs. Bioinformatics.

[74] Leigh J.W., Bryant D. (2015). POPART: Full-feature software for haplotype network construction. Methods Ecol. Evol..

[75] Clement M., Posada D., Crandall K.A. (2000). TCS: A computer program to estimate gene genealogies. Mol. Ecol..

[76] Excoffier L., Smouse P.E., Quattro J.M. (1992). Analysis of molecular variance inferred from metric distances among DNA haplotypes: Application to human mitochondrial DNA restriction data. Genetics.

[77] Churcher T.S., Schwab A.E., Prichard R.K., Basanez M.G. (2008). An analysis of genetic diversity and inbreeding in *Wuchereria bancrofti*: Implications for the spread and detection of drug resistance. PLoS Negl. Trop. Dis..

[78] Hedtke S.M., Zendejas-Heredia P.A., Graves P.M., Sheridan S., Sheel M., Fuimaono S., Lau C.L., Grant W.N. (2021). Genetic epidemiology of lymphatic filariasis in American Samoa after mass drug administration. Int. J. Parasitol..

[79] Rolland A., Balay G., World Health Organization, Onchocerciasis Control Programme in the Volta River Basin Area (1985). Onchocerciasis Focus in the Bissa Country.

[80] Ngoumou P., Walsh J.F., Ngoumou P., Walsh J.F. (1993). ; WHO Programme for the Prevention of Blindness. UNDP/World Bank/WHO Special Programme for Research and Training in Tropical Diseases. A Manual for Rapid Epidemiological Mapping of Onchocerciasis.

[81] Remme J.H.F., Boatin B., Boussinesq M., Quah S.R., Cockerham W.C. (2017). Helminthic diseases: Onchocerciasis and loiasis. The International Encyclopedia of Public Health.

[82] Duke B.O. (1991). Observations and reflections on the immature stages of *Onchocerca volvulus* in the human host. Ann. Trop. Med. Parasitol..

[83] Nelson G.S. (1991). Human onchocerciasis: Notes on the history, the parasite and the life cycle. Ann. Trop. Med. Parasitol..

[84] Prost A. (1980). Latence parasitaire dans l’onchocercose. Bull. World Health Organ..

[85] Hedtke S.M., Kuesel A.C., Crawford K.E., Graves P.M., Boussinesq M., Lau C.L., Boakye D.A., Grant W.N. (2020). Genomic epidemiology in filarial nematodes: Transforming the basis for elimination program decisions. Front. Genet..

[86] Koala L., Nikiema A., Post R.J., Paré A.B., Kafando C.M., Drabo F., Traoré S. (2017). Recrudescence of onchocerciasis in the Comoé valley in Southwest Burkina Faso. Acta Trop..

[87] Koala L., Nikiema A.S., Pare A.B., Drabo F., Toe L.D., Belem A.M.G., Boakye D.A., Traore S., Dabire R.K. (2019). Entomological assessment of the transmission following recrudescence of onchocerciasis in the Comoé Valley, Burkina Faso. Parasit. Vectors.

[88] Garms R. (1981). The reinvasion of the onchocerciasis control programme area in the Volta River Basin by *Simulium damnosum* s.l., the involvement of the different cytospecies and epidemiological implications. Ann. Soc. Belg. Med. Trop..

[89] Garms R., Walsh J.F., Davies J.B. (1979). Studies on the reinvasion of the Onchocerciasis Control Programme in the Volta River Basin by *Simulium damnosum* s.I. with emphasis on the south-western areas. Tropenmed Parasitol..

[90] Magor J.I., Rosenberg L.J. (1980). Studies of winds and weather during migrations of *Simulium damnosum* Theobald (Diptera: Simuliidae), the vector of onchocerciasis in West Africa. Bull. Entomol. Res..

[91] Baker R.H., Guillet P., Seketeli A., Poudiougo P., Boakye D., Wilson M.D., Bissan Y., Garms R., Cheke R.A., Sachs R. (1990). Progress in controlling the reinvasion of windborne vectors into the western area of the Onchocerciasis Control Programme in West Africa [and discussion]. Philos. Trans. R. Soc. Lond. B Biol. Sci..

[92] McCulloch K., Hedtke S.M., McCaw J., McVernon J., Basáñez M.-G., Walker M., Kuesel A.C., Grant W.N. (2023). Impact of human movement between hypo- and hyperendemic areas on sustainability of elimination of *Onchocerca volvulus* transmission. medRxiv.

[93] de Vos A.S., Stolk W.A., de Vlas S.J., Coffeng L.E. (2018). The effect of assortative mixing on stability of low helminth transmission levels and on the impact of mass drug administration: Model explorations for onchocerciasis. PLoS Negl. Trop. Dis..

[94] Specht S., Hoerauf A., Adjei O., Debrah A., Buttner D.W. (2009). Newly acquired *Onchocerca volvulus* filariae after doxycycline treatment. Parasitol. Res..

