# Supplementary material for: Assessing Onchocerca volvulus Intensity of Infection and Genetic Diversity Using Mitochondrial Genome Sequencing of Single Microfilariae Obtained before and after Ivermectin Treatment
Source: Pathogens. 2023 Jul 24;12(7):971. doi: 10.3390/pathogens12070971 (PMC10385737; doi:10.3390/pathogens12070971)
Supplement: Supplementary file 1 [file pathogens-12-00971-s001.zip › Supplementary Figure S1.pdf]

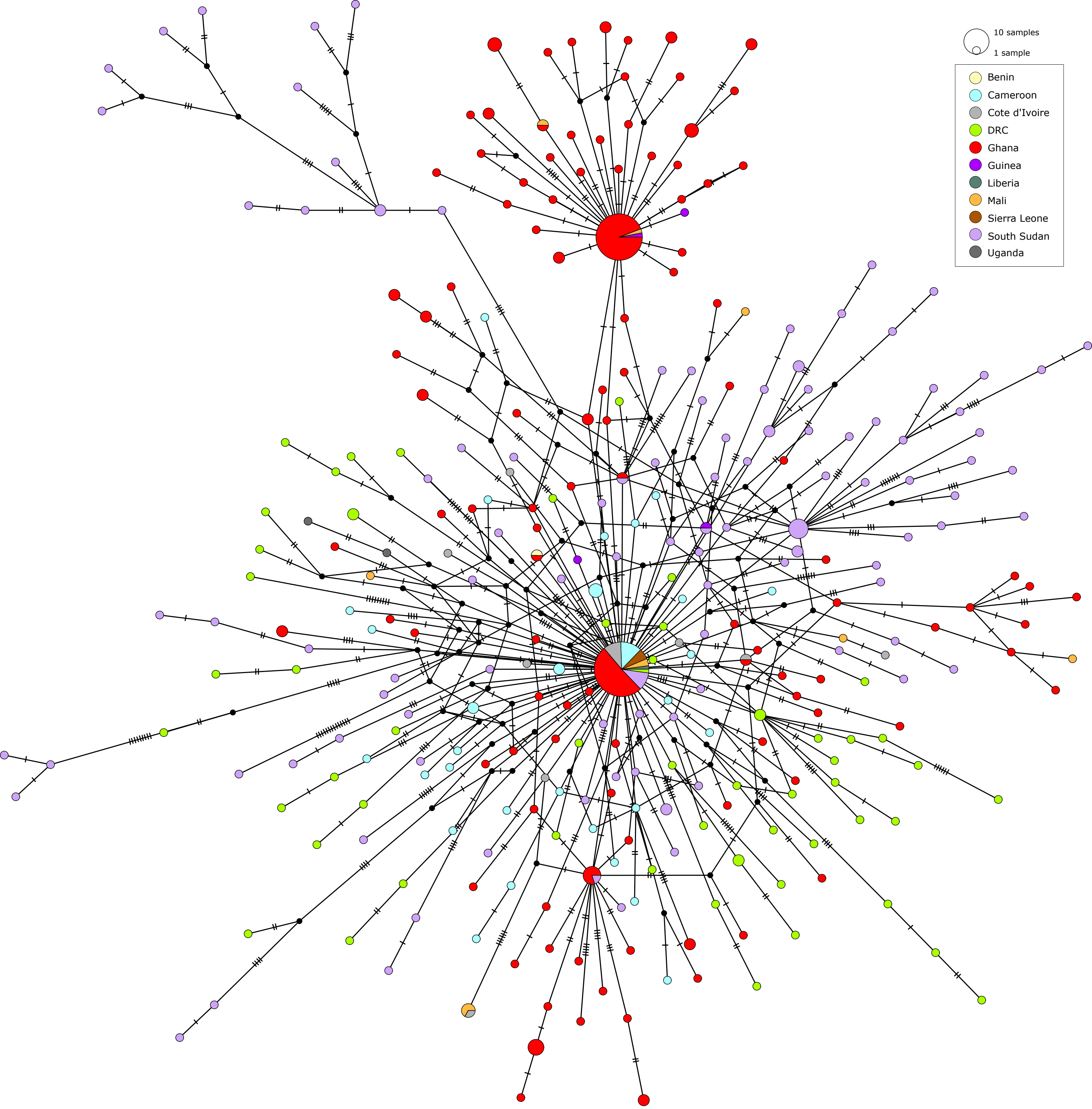

Figure S1. Haplotype network of mitochondrial haplotypes of *Onchocerca volvulus* collected in Benin, Cameroon, Côte d'Ivoire, Democratic Republic of Congo (DRC), Ghana, Guinea, Liberia, Mali, Sierra Leone, South Sudan, and Uganda. The size of the circles indicates the number of identical haplotypes; the number of hatch marks along network branches indicates the number of sequence differences between haplotypes; colors indicate the country of origin for each haplotype sampled.
